# Validity and reliability of myotonometry for assessing muscle viscoelastic properties in patients with stroke: a systematic review and meta-analysis

**DOI:** 10.1038/s41598-021-84656-1

**Published:** 2021-03-03

**Authors:** Maria-Isabel Garcia-Bernal, Alberto Marcos Heredia-Rizo, Paula Gonzalez-Garcia, María-Dolores Cortés-Vega, María Jesús Casuso-Holgado

**Affiliations:** Department of Physical Therapy, Faculty of Nursing, Physiotherapy and Podiatry, University of Sevilla, Sevilla, Spain

**Keywords:** Rehabilitation, Skeletal muscle, Cerebrovascular disorders, Stroke, Diseases of the nervous system, Quality of life, Therapeutics, Disability

## Abstract

There is a lack of consensus about the measurement of the muscle viscoelastic features in stroke patients. Additionally, the psychometric properties of the most-commonly used clinical tools remain controversial. Our objective is to investigate the validity and reliability of myotonometry to assess viscoelastic muscle features in stroke survivors. Pubmed, PEDro, Scopus and Cinahl were systematically searched to include studies reporting the psychometric properties of myotonometric devices used in people after stroke. The QUADAS-2 and the COSMIN checklists were used to assess the methodological quality of the studies and the psychometric properties of myotonometry. Nine studies were included in the qualitative synthesis and data from five of these were pooled in a meta-analysis. Overall, low to moderate risk of bias and applicability concerns were observed. Pooled data from intra-rater reliability for muscle tone showed a mean coefficient of correlation of 0.915 (95% CI: 0.880–0.940, I 2 = 69.2%) for upper limbs, and a mean coefficient of 0.785 (95%CI: 0.708–0.844, I 2 = 4.02%) for lower limbs. Myotonometry seems to be a valid and reliable complementary tool to assess muscle viscoelastic properties in stroke survivors, although definite conclusions about concurrent validity need further research.

## Introduction

Stroke is among the leading causes of death and disability-adjusted life years worldwide, and shows an increasing prevalence, with over 70 million stroke survivors expected by 2030^1^. Only in the European Union, the annual costs related to stroke treatment are estimated to be approximately 38 billion euros^2^.

In stroke patients, paresis is caused by a disruption of central motor command, which leads to stretch-sensitive (spastic) muscle overactivity, and adaptative changes in the viscoelastic properties of the soft tissues^3^. Spasticity is described as a velocity-dependent increase in muscle tone (hypertonia). It affects 4 to 27% of survivors in the acute or sub-acute phase and 17 to 42.6% in the chronic stage^4^, and is strongly associated with a reduced quality of life, loss of independence, and depression^5^.

The spastic hypertonia syndrome is currently understood as a multifactorial disorder, which accounts for biomechanical, histological and structural muscle changes^6^, such as shorter optimal fascicle length^7,8^, reduced muscle thickness^7^, and increased non-reflex stiffness or decreased muscle compliance, as a result of the adaptative muscle shortening^9^.

Muscle tone can be evaluated with non-instrumented, e.g., clinical scales, or instrumented tools, e.g., electrophysiological or mechanical measures. The original or modified Ashworth scale (MAS) and Tardieu scale (TS) are the most common non-instrumented measures in the clinical setting^10^. The Ashworth Scale (AS) and the MAS quantify the muscle response to an external movement, and they are used as a ‘gold standard’ to assess muscle tone in many studies^10^. Yet, these are subjective scales^11^, which tend to cluster^12^, and reveal little evidence of their psychometric properties^10^. The TS and the Modified Tardieu Scale (MTS) grade the muscle response to different stretching velocities^13^, and have demonstrated moderate to high reliability^10^, although these psychometric data are mainly related to patients with cerebral palsy^14^. As regards instrumented measures, a combination of neurophysiological and mechanical devices is the most recommended approach^10^. However, their clinical use is also controversial^15^ due to the lack of standardization of the measurement protocols and the need for costly equipment and specific training^16^. Therefore, cheap, objective, and easy-to-use devices are required in a clinical context.

Myotonometry represents a novel and non-invasive method to characterize the biomechanical and viscoelastic muscle properties^17^ such as compliance, stiffness, tone, elasticity, relaxation time and creep. Currently, there are two types of hand-held myotonometric devices. The Myotonometer^a^ measures the muscle deformation capacity in response to a number of repeated perpendicular forces (from 0.25 kg to 2 kg, at intervals of 0.25 kg) applied to the skin, during rest or isometric contraction, through a metal probe instrumented with a linear range of transducers^18^. Muscle compliance is derived from the slope of the muscle displacement-force function, as an inverse of stiffness^18^. The MyotonPRO^b^, an improved version of the Myoton 3^b^, applies a brief mechanical impulse to the skin to record different oscillation parameters of the muscle response^19^. Dynamic stiffness refers to the soft tissue resistance to an external force and is calculated using the damped natural oscillation response, as registered by an in-built accelerometer^20^. Muscle tone is quantified by the natural frequency of the acceleration signal, while muscle elasticity, which is inversely proportional to the decrement, is determined by the sequential oscillations when the muscle restores its shape from deformation^21^. Stress relaxation time reflects the duration of the muscle recovery process^21^, and muscle creep is defined as the gradual elongation of the muscle under a constant tensile stress^22^. In healthy participants, the Myotonometer^a^ demonstrates moderate to good intra- and inter-rater reliability, and appears not to be valid for the measurement of stiffness^23^. The MyotonPRO^b^ has been validated for measurements of elasticity^24^ and stiffness^20^ in healthy participants, and reports good to excellent intra- and inter-rater reliability in healthy skeletal muscles^25^. To date, this is the first study aiming to summarize the available evidence regarding the psychometric properties of myotonometry in patients after stroke. This systematic review investigates the validity and reliability of myotonometry to evaluate upper and lower limb muscle viscoelastic properties in post-stroke patients, and aims to describe the assessment protocol with the highest quality evidence.

## Methods

This systematic review was performed following Preferred Reporting Items for Systematic Reviews and Meta-Analyses (PRISMA) recommendations and was registered in the PROSPERO database (CRD42018105751).

### Research question and study selection

The research question for this systematic review was: Are the myotonometric devices valid and reliable tools for the assessment of the muscle viscoelastic properties in post-stroke patients? Thus, inclusion criteria for this review were as follows:

#### Participants

To be included, participants had to: (1) be adults aged over 18 years, (2) have been clinically diagnosed with a first-event stroke according to the current World Health Organization definition^26^.

#### Studies

This review was restricted to studies aiming to evaluate the psychometric properties of different myotonometric devices. The reference standard for validity studies had to be commonly used muscle property assessment tools. Self-reported scales were excluded. Participants had to: (1) be adults aged over 18 years, (2) have been clinically diagnosed with a first-event stroke according to the current World Health Organization definition. Finally, to be included, studies had to evaluate at least one muscle viscoelastic property of interest in adults with a stroke (e.g., tone, stiffness, compliance, or elasticity). Only published papers written in English were admitted at this stage. No publication date restriction was established. As for exclusion criteria, studies not using myotonometric devices as an assessment tool were excluded.

### Data sources and search strategy

The following electronic databases were searched: Pubmed, PEDro, Scopus and Cinahl, starting in December 2018 and finishing on June 2019.

The search strategy included all available records with the following Medical Subject Heading Terms: Musc* tone or Spastic* or Musc* Stiffness or Musc* Propert* AND Valid* or Reliab* or Reproducib* or Accura* or Psychometr* AND Stroke or Brain Injur* or Cerebrovascular Accident* or Hemipleg* or Hemipares* or Aploplex* (Table S1). Myoton* or tonomet* were not included as descriptors in the search strategy to avoid exclusion of papers using devices of similar mechanism but different names.

### Data extraction and quality assessment

First, articles were selected by screening of the title and abstract by two independent reviewers (PGG and AMHR) and duplicates were removed. The full text of eligible records were thoroughly read to determine whether the inclusion/exclusion criteria were met. Any doubt was discussed and solved with the help of other researcher (MJCH).

When articles suitable for inclusion in this research were identified two reviewers (AMHR and MJCH) independently extracted the following information from each study using a standardized form: measured psychometric properties of myotonometric devices; characteristics of participants (e.g., sample size and study groups, mean age, sex distribution, and mean time post-stroke); assessed muscles; myotonometric tool and outcome measures; reference standard for validity studies; raters and timing of measurements for reliability studies; evaluation protocol; and main results.

The quality of studies regarding risk of bias and applicability concerns was measured using the Quality Assessment of Diagnostic Accuracy Studies-2 (QUADAS-2) tool^27^, which it is recommended by the Cochrane Collaboration for critical appraisal of research on diagnostic test accuracy^28^. The QUADAS-2 tool lists four main domains: patient selection, index test, reference standard, and flow and timing^27^. The reference standards differed among the included studies, hence the use of myotonometry was compared to several stated measures of mechanical, viscoelastic and functional properties of the muscle. Following previous guidelines^29^, no article was excluded based on the selected reference standard. We also used the COnsensus-based Standards for the selection of health status Measurement Instruments (COSMIN) Risk of Bias checklist^30,31^. Boxes 6 to 10 from the checklist were chosen, whenever applicable, to assess risk of bias regarding reliability, measurement error, criterion validity, hypothesis testing for construct validity and responsiveness. This newly updated checklist has been developed from the original COSMIN^32^, to be used exclusively in systematic reviews of measurement properties of patient reported outcome measures. Every item is ranked on a 4-point scale from very good to inadequate, and the lowest rating of any item within a box is used to determine the risk of bias (“worse score counts”)^30^. The two reviewers independently rated the quality of studies and, if necessary, a final consensus was reached in the case of disagreement.

### Data synthesis and meta-analysis

The results for criterion and construct validity and reliability were described and the overall clinimetric evidence of the myotonometric devices was summarized. Where possible, reliability studies providing similar data (e.g., intra or inter-rater reliability for lower or upper limbs muscles) were pooled and the meta-analysis was conducted assuming a random-effects model. Data from overlapping samples were screened to avoid bias in the quantitative analysis.

For criterion validity, the strength of correlations was interpreted as low (< 0.25), fair (0.25–0.50), moderate to good (0.50–0.75) and good to excellent (> 0.75)^33^. For relative reliability interpretation, Intraclass Correlation Coefficients (ICCs) scores of less than 0.4 were considered poor, 0.4 to 0.59 as fair, 0.6 to 0.74 as good and > 0.75 as excellent^34^. For absolute reliability interpretation, a standard error of measurement (SEM) < 10% was considered small; standard real difference (SRD) was considered acceptable when < 30% and excellent when < 10%, and the narrower limits of agreement (LOA) indicates a higher stability. These benchmarks were established in accordance with previous research^35^ because quantification of absolute reliability statistics is controversial^36^. Comprehensive Meta-Analysis version 3.0 software was used to construct forest plots, and Review Manager version 5.0 was used for risk of bias and applicability concerns summary graph.

## Results

### Study selection

**Figure 1:**
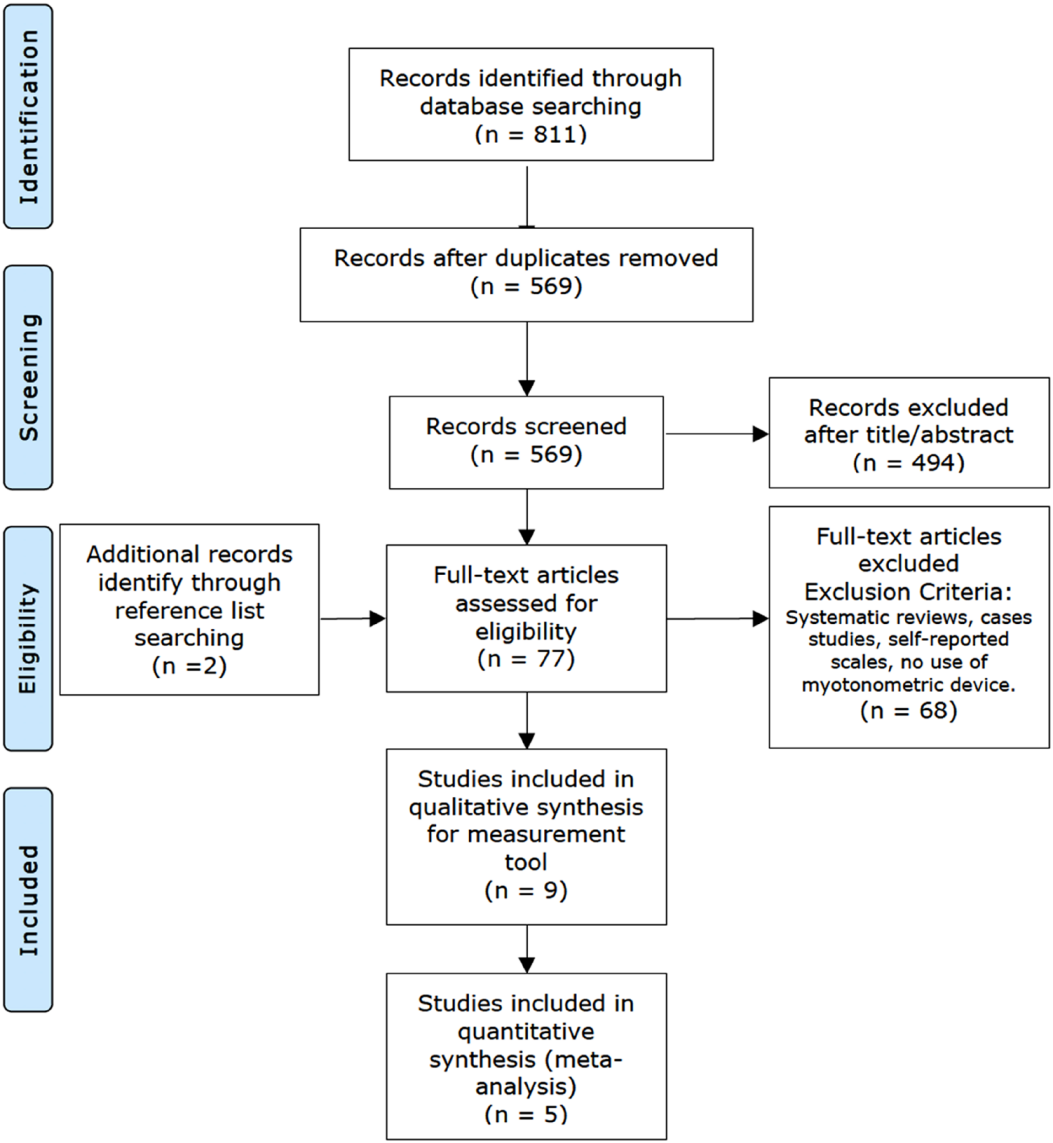
Flow diagram of trial selection based on PRISMA guidelines.

The PRISMA flow diagram (Fig. 1) shows the screening process followed to reach the final studies analyzed in this review. The initial electronic search identified 811 records, most of which were discarded at different stages. Two additional records were identified within the reference lists of other articles. Nine studies meeting the inclusion criteria were included in the qualitative synthesis^37–45^. For the quantitative analysis, three of them were excluded due to the heterogeneity in the reference standard test used^43–45^ whereas another paper was excluded because was the only one that investigated inter-observer reliability^38^. Therefore, only five studies were included in the meta-analysis^37,39–42^.

### Methodological quality assessment

**Figure 2:**
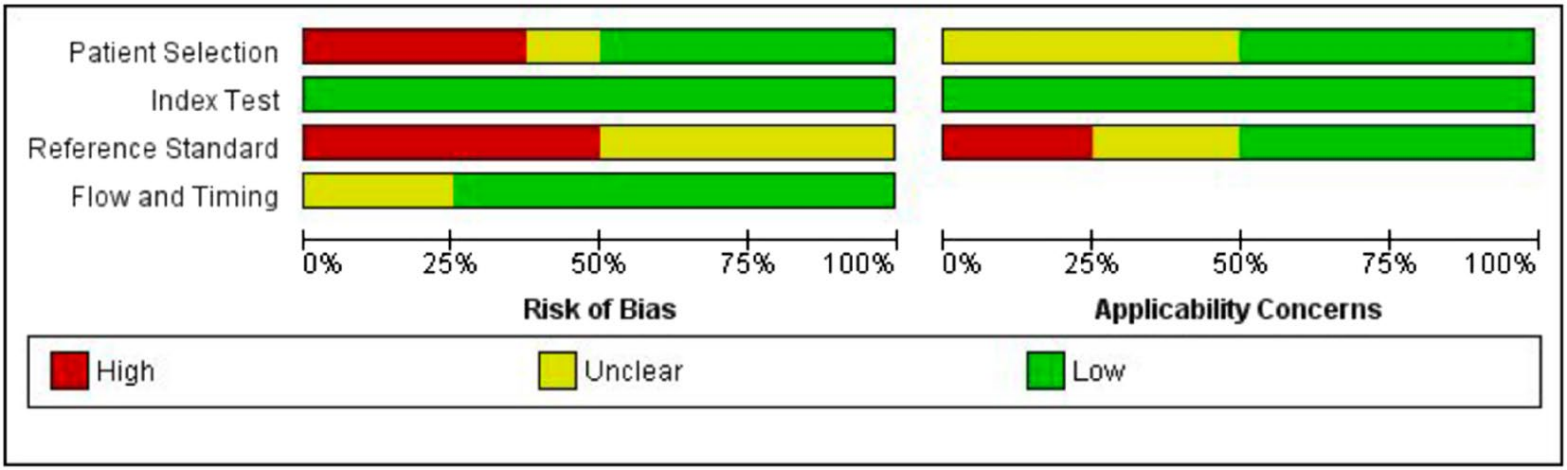
Risk of bias and applicability concerns graph.

Figure 2 and Table S2 illustrate the assessment of methodological quality, based on the QUADAS-2 tool, in terms of risk of bias and applicability concerns. Criterion validity studies^40,42–45^ were assessed for all domains, whereas the rest of them were only evaluated for patient selection^37–39,41^. No single study showed a low risk of bias on all domains. Yet, all assessed studies used an acceptable protocol for the index test, except for Ryhdal et al.^45^, where the index test was conducted with knowledge of the results of the reference standard. The risk of bias was high in three studies for patient selection^39–41^, and in two studies for the reference standard, which was interpreted from the results of muscle strength and/or function^40,42^.

The COSMIN critical appraisal checklist (boxes 6 to 10) was applied to all nine studies (Tables S3–S8). Six reported the reliability and measurement error of the different tools, and were assessed as doubtful^37–41^ or inadequate^40,42^ methodological quality. Five studies included criterion validity, which inconsistently rated very good^42,44^, doubtful^40,45^ or inadequate methodological quality^43^. The three studies evaluating the construct validity of the Myotonometer^a^ or the MyotonPRO^b^ rated as having an adequate^45^, inadequate^43^ or doubtful^42^ methodological quality. Finally, a single study reported responsiveness and was considered with inadequate quality^40^.

### Study design and population characteristics

For validity analysis, a sample of 188 first stroke survivors^40,42–45^ (30.31% women, mean age 56.53 years) in the chronic stage, mean time after stroke 32.02 months^40,42,43,45^ , were included together with 54 healthy controls (44.44% women, mean age 57.5 years)^42,43,45^. All the subjects were chronic stroke survivors (mean time post-stroke of 32.02 months). For reliability analysis, a sample of 121 individuals (35.53% women, mean age 54.29 years) was analyzed^38,39,41,42^. Within this sample, 80 subjects were in the chronic stage (mean time post-stroke of 22.11 months)^41,42^, 12 were in the sub-acute phase (3 to 9 months post-stroke)^39^ and the remaining 29 were in the acute stage (< 1 month post-stroke). The average sample size was 39.6 subjects (range 14 to 67) for validity studies and 34.5 (range 12 to 61) for reliability.

Three different myotonometric devices were included: Myotonometer^a^^43–45^, Myoton-3 myometer^b^^39–41^ and MyotonPRO^b^^37,38,42^. A detailed description of the study characteristics and main results for validity for upper and lower limbs are reported in Tables 1 and 2, respectively; and Tables 3 and 4 for reliability.

**Table 1:** Main characteristics of selected studies analyzing validity of the myotonometry in the upper limbs.

Study	Participants	Tool and muscles	Reference standard	Assessment protocol	Main results
Chuang et al.^40^	N = 67 67 chronic stroke patients Mean age = 54.67, SD = 10.9 27 women Mean time post stroke (mo) = 21.12, SD = 13.63	Myoton-3 myometer: Muscle tone, stiffness, and elasticity at rest MB: Extensor digitorum; Flexor carpi radialis Flexor carpi ulnaris	1. Hydraulic hand dynamometer and pinch gauge: Hand strength (grip, lateral pinch and palmar pinch) 2. ARAT test: Arm function	Relaxed supine with 30° to 45° elbow flexion, palm downward for the extensor digitorum and palm upward for flexor carpi radialis and ulnaris (myoton-3 myometer); seated, shoulder adducted and neutrally rotated, 90° elbow flexion, and forearm and wrist in neutral position (hydraulic dynamometer and pinch gauge) 1. Myoton-3 myometer: 3 bilateral trials at rest, 1 s interval. Left side first 2. Hydraulic hand dynamometer and pinch gauge: 3 bilateral trials, 2 to 3 min interval. Left side first 3. ARAT test	Low to fair correlations between myometer scores, hand strength and arm function No general concurrent patron between outcome measures
Leonard et al.^43^	N = 20 10 with UMN disorders (6 with stroke and 4 with cerebral palsy) Mean age = 47.5 4 women Mean time post stroke = N/A 10 healthy controls Mean age = 48.3 4 women	Myotonometer: Muscle compliance at rest and during MVC MB: Biceps brachii	1. MAS test of the elbow flexor muscles: Muscle tone	Sitting position with 90° elbow flexion and forearm supinated (myotonometer and MAS) 1. Myotonometer: 5 trials at rest and 5 trials during MVC of the biceps brachii. 15 to 30 s rest between trials. Force intervals of 2.5 N over a range from 2.5 to 20 N Bilateral assessment for subjects with UMN disorders, and on the dominant side for controls 2. MAS test	Moderate to high correlations (0.64–0.81) between MAS scores and percentage differences of the myotonometer (relax vs. MVC)
Li et al.^44^	N = 14 14 chronic stroke patients Mean age = 61 8 women Mean time post stroke (mo) = 61, SD = 30	Myotonometer: Muscle compliance at rest MB: Biceps brachii	1. MAS test of the elbow flexor muscles: Muscle tone 2. Conventional muscle stretch test with a torque sensor: Reflex and non-reflex elbow flexor torque	Sitting position with 90° elbow flexion and shoulder slightly abducted (myotonometer); and 45° shoulder abduction with 30° shoulder flexion (stretch test) 1. Myotonometer: 8 bilateral trials at rest. Force intervals of 2.5 N over a range from 2.5 to 20 N 2. Muscle stretch test: Ramp and hold protocol. 2 s rest, constant velocity stretch of elbow flexors, 2 s holding pause, return to initial position. 50° of total range of stretch at 5°/s or 100°/s. 3 trials with 30 s for each velocity	Reductions in muscle compliance and AUC between the spastic and non-spastic sides Negative moderate association between muscle compliance and AUC and the stretch test at 100°/s No correlations between MAS scores and muscle compliance

ARAT: Action Research Arm Test; AUC: area under the curve; N: newton; N/A: non available; MB: muscle belly; mo: months; MVC: maximal voluntary contraction; UMN: upper motor neuron.

**Table 2:** Main characteristics of selected studies analyzing validity of the myotonometry in the lower limbs.

Study	Participants	Tool and muscles	Reference standard	Assessment protocol	Main results
Fröhlich-Zwahlen et al.^42^	N = 40 20 chronic stroke patients Mean age = 52, SD = 11 9 women Mean time post stroke (y) = 1.9, SD = 0.7 20 healthy controls Mean age = 53, SD = 10 9 women	MyotonPRO: Muscle tone, stiffness, and elasticity at rest MB: Vastus lateralis Rectus femoris TA Biceps femoris GM Medialis	1. US: Muscle and subcutaneous tissue thickness 2. Dynamometer: Isometric strength (MVC) of the knee extensor and flexor, plantar flexor and dorsiflexor muscles	Relaxed supine and prone position (MyotonPRO and US), standard seated position (dynamometer) 1 h session 1. MyotonPRO: 10 single trials at rest in each muscle. Impulse force 0.4 N of 15 ms duration. 1 s interval between trials 2. US: 2 longitudinal or transverse pictures with a short break 3. Dynamometer: 3 trials of 5 s duration. 30 s interval between trials	Low to fair correlations between muscle strength or muscle thickness and muscle tone, stiffness and elasticity Group effect for stiffness of the medialis GM
Rydahl & Brouwer^45^	N = 47 23 chronic stroke patients Mean age = 67.5, SD = 10.9 9 women Mean time post stroke (y) = 4.6, SD = 3.3 24 healthy controls Mean age = 71.2, SD = 9 11 women	Myotonometer: Muscle compliance at rest and during 10% MVC MB: GM	1. MAS test of the plantar flexor muscles: Muscle tone 2. Torque motor and electric stimulator: Stiffness of ankle muscles (total, passive, intrinsic and reflex stiffness)	Seated with lower limbs hanging (MAS test), semi-reclined position with test leg in a support frame, 90° between trunk and hip, and 45° knee flexion (torque motor and myotonometer) 1 h session 1. MAS test 2. Torque motor: 1 kHz per channel over 1050-ms, with 500-ms pre- and post perturbation. 5 trials for condition: rest, voluntary 10% of MVC, and involuntary 10% of MVC (electric stimulation of the posterior tibial nerve in the popliteal fossa for less than 5 s, with 1-ms square-wave pulses at 30 Hz. Perturbation of 5° at 100°/s in all conditions 3. Myotonometer: 3 trials at rest, and 3 trials during 10% of MVC. Force intervals of 2.5 N over a range from 2.5 to 20 N	Differences in compliance between rest and 10% MVC were negatively correlated with higher MAS scores and total ankle stiffness. Low to fair association observed No main group effect for muscle compliance at rest or during 10% VC Differences between stroke patients and controls in muscle compliance for the AUC, and the percentage difference between rest and contracted conditions

AUC: area under the curve; GM: gastrocnemius muscle; N: newton; MB: muscle belly; MVC: maximal voluntary contraction; TA: tibialis anterior; US: Ultrasonography.

**Table 3:** Main characteristics of selected studies analyzing reliability of the myotonometry in the upper limbs.

Study	Participants	Tool and muscles	Assessment protocol	Main results
Lo et al.^37^	N = 28 28 acute (< 1 mo) stroke patients Mean age = 58.7, SD = 12.8 4 women Mean time post stroke (days) = 22, SD = 7.36	MyotonPRO: Muscle tone at rest MB: Biceps brachii, Brachio-radialis	Supine lying position, with 10 to 15° elbow flexion and forearm supinated (biceps brachii), or elbow extension and forearm pronated (brachioradialis) Evaluation of affected side. A set of 3 impulses, with 1 s rest interval, 1 day between tests	Excellent reliability (ICCs = 0.75–0.82), and no systematic bias between measurements Muscle tone: SEM: 0.50 to 0.76; SRD: 1.96 to 2.41; 95% LOA: + 3.4 to -3.17
Lo et al.^38^	N = 29 29 acute (< 1 mo) stroke patients Mean age = 58.9, SD = 12.6 5 women Mean time post stroke (days) = 20, SD = 7.16	MyotonPRO: Muscle tone, stiffness, decrement and creep at rest MB: Biceps brachii, Brachio-radialis	Supine lying position, with 10 to 15° elbow flexion and forearm supinated (biceps brachii), or elbow extension and forearm pronated (brachioradialis) Bilateral evaluation. A set of 3 impulses, with 1 s rest interval, 15 min between tests	Moderate to very high reliabilities (ICCs = 0.65–0.93) for affected and unaffected sides Muscle tone: SEM: 2.85% to 3.73%; SRD: 6.65% to 8.71%; 95% LOA: + 2.23 to -2.61/ Muscle stiffness: SEM: 4.21% to 6.18%; SRD: 9.82% to 14.43%; 95% LOA: + 54.17 to -70.597/Muscle creep: SEM: 4.39% to 5.71%; SRD: 10.23% to 13.32%; 95% LOA: + 0.27 to -0.28
Chuang et al.^39^	N = 12 12 sub-acute (3 to 9 mo post-stroke) stroke patients Mean age = 51.19, SD = 11.02 4 women Mean time post stroke (mo) = 6.58, SD = 1.38	Myoton-3 myometer: Muscle tone, stiffness, and elasticity at rest MB: Biceps brachii, Triceps brachii	Supine position (biceps brachii), and side lying (triceps brachii), with arms at the sides, and forearms between pronation and supination Bilateral evaluation (unaffected side first), 3 sets with 1 s rest interval, 60 min between tests	Excellent reliability (ICCs = 0.79–0.96), except for biceps brachii muscle tone on the unaffected side (ICC = 0.72) Muscle tone: SEM: 2.93% to 8.04%; MDC90: 6.82% to 18.67%/Muscle stiffness: SEM: 4% to 10.72%; MDC90: 9.31% to 24.98%/Muscle elasticity: SEM: 6.04% to 7.26%; MDC90: 13.42% to 16.75%
Chuang et al.^40^	N = 58 58 chronic stroke patients Mean age, Mean time post stroke = N/A	Myoton-3 myometer: Muscle tone, stiffness, and elasticity at rest MB: Extensor digitorum, Flexor carpi radialis and ulnaris	Relaxed supine position, Bilateral evaluation (left side first): 3 sets, with 1 s rest interval, 30 min between tests	High to very high reliability (ICCs = 0.75–0.96) in the affected and unaffected sides
Chuang et al.^41^	N = 61 61 chronic stroke patients Mean age = 55.08, SD = 10.9 25 women Mean time post stroke (mo) = 21.43, SD = 14.04	Myoton-3 myometer: Muscle stiffness, tone and elasticity at rest MB: Deltoid, Triceps brachii Biceps brachii	Relaxed supine position, elbow extended, forearm supinated and arm placed on the table (biceps brachii), and relaxed side lying position with the arm at the side and forearm between pronation and supination (deltoid and triceps brachii) Evaluation of affected side, 3 sets, with 1 s rest interval, 30 min between tests	High to very high relative and absolute reliabilities (ICCs = 0.86–0.94) Muscle tone: SEM: 3.9% to 6.7%; SRD: 10.7% to 18.7%; LOA: + 4.37 to -4.49/ Muscle stiffness: SEM: 4.3% to 6%; SRD: 12% to 16.5%; LOA: + 74.60 to -82.28/ Muscle elasticity: SEM: 4.9% to 8.9%; SRD: 13.5% to 24.4%; LOA: + 0.61 to -0.65

N/A: non available; MB: muscle belly; MDC: minimal detectable change; mo: months; SRD: smallest real difference.

**Table 4:** Main characteristics of selected studies analyzing reliability of the myotonometry in the lower limbs.

Study	Participants	Tool and muscles	Assessment protocol	Main results
Lo et al.^37^	As previously reported for upper limbs	MyotonPRO: Muscle tone at rest MB: Rectus femoris, TA	Supine lying position, with hips neutral and full knee extension Evaluation of affected side, A set of 3 impulses, with 1 s rest interval, 1 day between tests	Excellent reliability (ICC = 0.81) and bias towards overestimating rectus femoris tone on the 1st measurement Muscle tone: SEM: 0.83 to 1.24; SRD: 2.52 to 3.08; 95% LOA: + 4.09 to -4.95
Lo et al.^38^	As previously reported for upper limbs	MyotonPRO: Muscle tone, stiffness, decrement and creep at rest MB: Rectus femoris, TA	Supine lying position, with hips neutral and full knee extension Bilateral evaluation, A set of 3 impulses with 1 s rest interval, 15 min between tests	Moderate to very high reliabilities (ICCs = 0.65–0.99) for affected and unaffected sides Muscle tone: SEM: 2% to 4.92%; SRD: 4.68% to 11.47%; 95% LOA: + 3.85 to -3.19/ Muscle stiffness: SEM: 2.16% to 6.18%; SRD: 5.05% to 14.41%; 95% LOA: + 88.40 to -81.36/ Muscle decrement: SEM: 4.02% to 14.03%; SRD: 9.38% to 32.73%; 95% LOA: + 0.76 to -0.82/ Muscle creep: SEM: 3.05% to 5.67%; SRD: 7.13% to 13.23%; 95% LOA: + 0.38 to -0.39
Fröhlich-Zwahlen et al.^42^	N = 19 19 chronic stroke patients Mean age = 52, SD = 11 9 women Mean time post stroke (y) = 1.9, SD = 0.7	MyotonPRO: Muscle tone, stiffness, and elasticity at rest MB: Vastus lateralis, Rectus femoris, TA, Biceps femoris GM Medialis	Relaxed supine/ prone position Evaluation of affected side, 10 single trials at each site, with 1 s rest interval, using an impulse force of 0.4 N with 15 ms duration, 7 days between tests	Moderate to high reliability (ICCs = 0.62–0.92) and low standard error measurements (-3.00 to 19.84) Muscle tone: SEM: 6% to 8.4%; Muscle stiffness: SEM: 5.1% to 9.2%; Muscle elasticity: SEM: 9.5% to 15.1%

GM: gastrocnemius muscle; N: newton; MB: muscle belly; MDC: minimal detectable change; SRD: smallest real difference; TA: tibialis anterior.

### Validity

#### Criterion validity

Five of the studies^40,42–45^ measured criterion validity of myotonometric devices by comparison with other muscle property assessment tools. Different tests were used as the reference or gold standard. The MAS was the most commonly used reference test for the spastic condition assessment^43,45^. Dynamometry (muscle strength) was also used for comparison in two trials^40,42^, as well as muscle stretching tests with a torque motor^44,45^. In addition, Fröhlich et al.^42^ evaluated muscle and subcutaneous tissue thickness with an ultrasound, while Chuang et al.^40^ assessed arm functionality.

All studies collected data on the same patients at the same time with an appropriate interval between tests. Clear evaluation protocols were defined in all cases; myotonometric tests were performed at the resting muscle condition^40,42,44^, or during rest and voluntary muscle contraction^43,45^. Myotonometry was always conducted and interpreted prior to the reference standard except for by Rydahl and Brouwer^45^.

Data analysis was based on Pearson´s correlations (r)^40,42,44,45^, Spearman coefficients (ρ)^4045^ and Cramer´s V correlation^43^. Results from correlation analysis found moderate to high correlations (V = 0.64–0.81) between MAS scores and percentage differences of compliance at rest and muscle contraction^43^; low to fair correlations (ρ = 0.412–0.453) between MAS and differences of compliance at rest and 10% maximal voluntary contraction^45^, and no correlations between MAS and muscle compliance^44^. Similarly, low to fair correlations (r = 0.30–0.40) were reported between myotonometry scores and muscle strength^40,42^. When myotonometer values were correlated with muscle stretching tests, a negative association between muscle compliance and the area under the curve and the stretch stiffness at 100º/s were observed (r = − 0.556, − 0.607, respectively)^44^. In contrast, low to fair associations (r = 0.436–0.542) were obtained between differences in muscle compliance and muscle stiffness^45^.

Finally, low to fair correlations (r = 0.29–0.46) were also observed between myotonometry scores and muscle thickness^44^, and arm function (r = 0.27–0.30)^40^.

#### Construct validity

Three studies assessed construct validity of myotonometry^42,43,45^. These trials aimed to determine if the devices could discriminate between healthy subjects and stroke patients, and between the involved and uninvolved extremities. Data analysis was based on analysis of variance (ANOVA).

Side effects were only observed for biceps brachii compliance (p: range, 0.03–0.05)^43^. The effect of group was significant for the stiffness of gastrocnemius medialis, which was higher in patients than in controls (p < 0.05), but not for all the other lower limb muscles and parameters^42^. For muscle compliance, scores for control and stroke groups were similar at rest^43,45^. When data was obtained during maximal voluntary contraction, significant differences (p: range, 0.01–0.00) between stroke and control participants were observed for the biceps brachii^43^, but not for the gastrocnemius medialis^45^. Yet, evaluation of the percentage difference in compliance (rest/contraction) revealed a significant difference between participants with and without stroke (p < 0.05) with smaller differences observed in those with stroke^43,45^ (Tables 3 and 4).

### Reliability

For studies investigating reliability^37–42^, five of them exclusively assessed intra-rater reliability^37,39–42^, and only one assessed inter-rater reliability^38^*.* The length of time between testing sessions ranged from 15 min^38^ up to seven days^42^. All studies investigated the reliability of myotonometry for muscle tone assessment. In addition, five studies explored the reliability of stiffness and elasticity/decrement assessment^38–42^, whereas muscle creep was an outcome measure in only one trial^38^.

**Figure 3:**
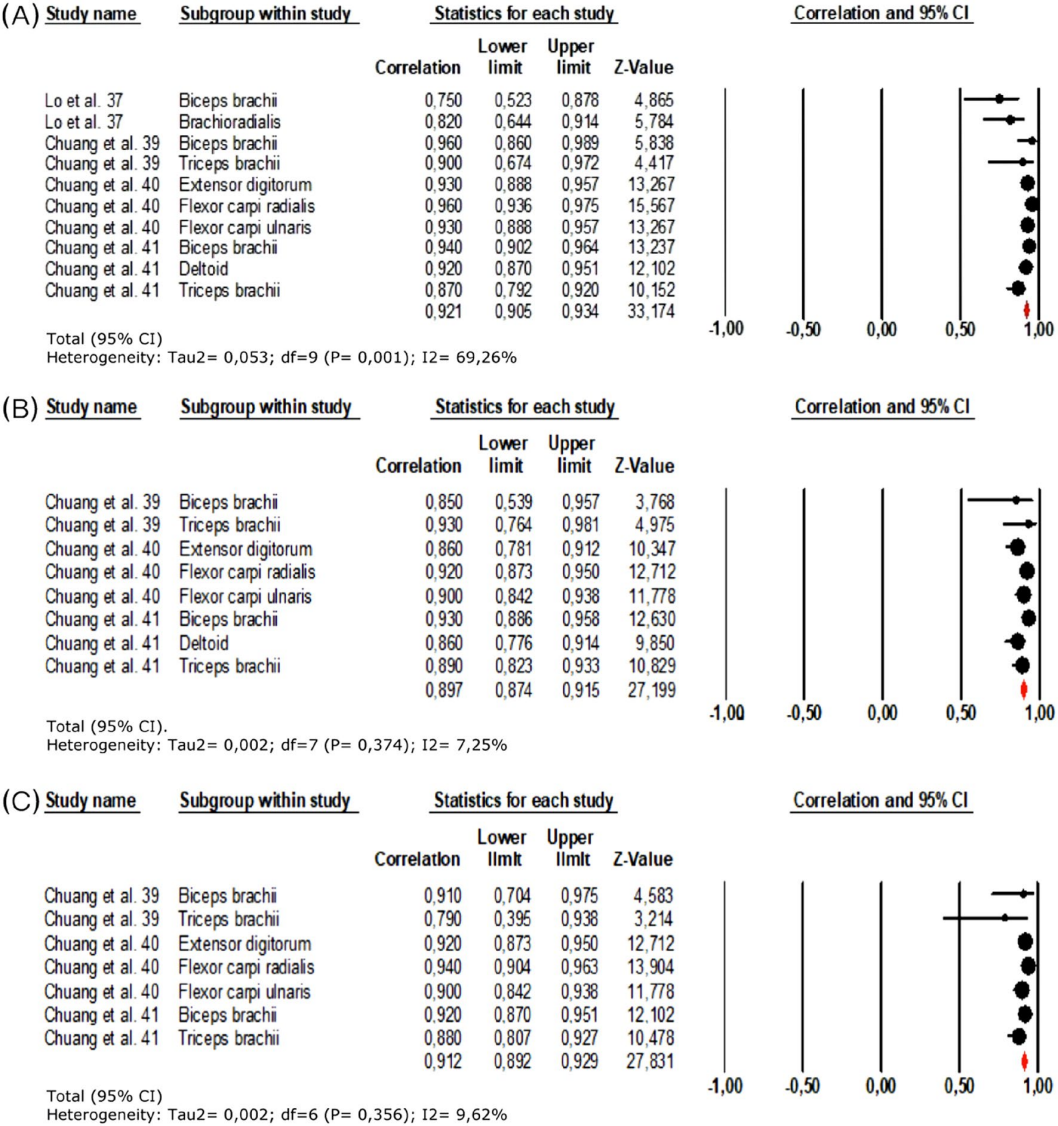
Forest plot of the ICC obtained from intra-rater reliability for upper limbs: (**A**) muscle tone, (**B**) muscle elasticity, (**C**) muscle stiffness.

**Figure 4:**
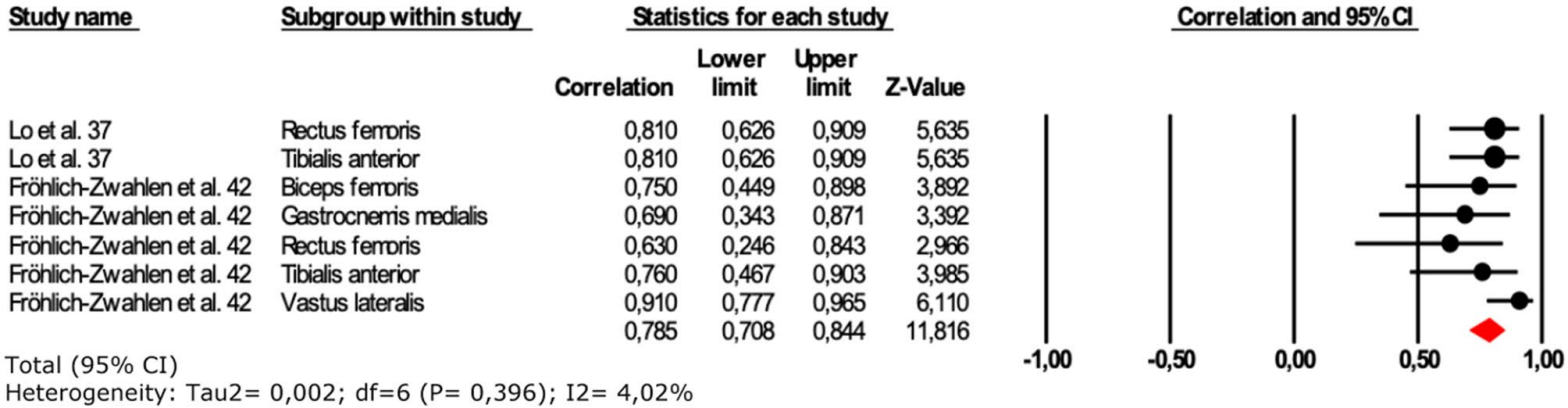
Forest plot of the ICC obtained from intra-rater reliability for lower limbs muscle tone.

The ICC was commonly used as a statistical test for the evaluation of reliability. All studies, except for Fröhlich-Zwahlen et al.^42^, reported excellent intra-rater reliability, with results ranging from ICC = 0.72 to 0.96 for the upper limbs, and ICC = 0.62 to 0.92 for the lower limbs. When the data from intra-rater reliability for the upper limbs were pooled, it exhibited a mean coefficient of correlation of 0.915 (95% Confidence Interval (CI): 0.880–0.940, I^2^ = 69.26%) for muscle tone (Fig. 3A), 0.897 (95%CI: 0.874–0.915, I^2^ = 7.25%) for muscle elasticity (Fig. 3B) and 0.912 (95%CI: 0.89–0.93, I^2^ = 9.62%) for muscle stiffness (Fig. 3C). Pooled data for intra-rater reliability for lower limb muscle tone reported a mean coefficient of 0.785 (95%CI:0.708–0.844, I^2^ = 4.02%) (Fig. 4). A single study^38^ measured inter-rater reliability, with the results showing moderate to very-high reliability (ICC = 0.65 to 0.93 for the upper limbs, and ICC = 0.65 to 0.99 for the lower limbs). There were no differences for intra or inter-rater reliability of the evaluation of the affected or unaffected sides^38–40^. Five studies reported SEM percentages^37–39,41,42^, which ranged from 2 to 8.04% for muscle tone, 2.16 to 10.72% for muscle stiffness, 4.02 to 15.1% for muscle decrement/elasticity, and 3.05 to 5.71% for muscle creep. The SRD values were measured in three studies^37,38,41^ and varied from 6.65 to 18.7% for muscle tone, 9.82 to 16.5% for muscle stiffness, 13.5 to 24.4% for muscle decrement/elasticity, and 10.23% to 13.32% for muscle creep^40^. The percentages for the minimal detectable change ranged from 6.82 to 24.98% and were reported in one study^39^. Finally, the 95% LOA were evaluated in three studies^37,38,41^, and varied between + 4.37 to − 4.95 for muscle tone, + 88.40 to − 82.28 for muscle stiffness, + 0.76 to − 0.82 for muscle decrement/elasticity, and + 0.38 to − 0.39 for muscle creep. The high variability between studies for SEM, SRD and minimal detectable change percentages, and the 95% LOA, could be attributable to the different myotonometric devices used and the diversity of the assessment protocols (Tables 3 and 4).

## Discussion

This systematic review and meta-analysis aimed to summarize the risk of bias and the findings of studies evaluating the psychometric properties of myotonometric devices used for the assessment of muscle viscoelastic properties related to the spastic hypertonia syndrome in stroke patients. A total of nine studies were included in the qualitative synthesis, and data from five of them were pooled in a meta-analysis. In general, low to moderate risk of bias and applicability concerns were observed. The following three reasons may account for lower scores: (1) no severe spasticity condition as inclusion criteria (MAS ≤ 2)^39–41^, which could be considered as a patient selection bias; (2) inappropriate time interval between measurements for reliability assessment^38,42^ and (3) the use of reference standards that are not likely to correctly classify the target condition^40,42^.

When criterion validity was analyzed, low to fair correlations were observed between muscle compliance, assessed with the Myotonometer^a^, and the MAS scores^43–45^. These results are similar to those observed by Drenth et al.^46^, who analyzed the correlations between the MyotonPRO^b^ scores on the biceps brachii and the MAS for Paratonia. In this former study, no correlation was reported for elasticity, and poor associations were observed for muscle tone, stiffness and creep (ρ < 0.5). Similarly, low to fair correlations were reported between muscle strength and muscle tone, stiffness and elasticity when assessed with the Myoton-3^b^^40^ or the MyotonPRO^b^^42^. In agreement with the present findings for criterion validity of myotonometry in the assessment of spasticity, Bar-On et al.^47^ observed poor correlations between the electrophysiological findings of the instrumented tests and the MAS scores. These findings were interpreted as a confirmation of the inadequacy of the clinical tests. Poor correlations were also obtained between the percentage differences in compliance between rest and muscle contraction and the total ankle stiffness assessed by a torque motor system^45^. Notwithstanding, Rydahl and Brouwer pointed out that stronger correlations could have been obtained if each muscle could be stretched in isolation^45^. These slight correlations are congruent with previous considerations for the measurement of spasticity. Previous research highlights that data obtained from the measurement of muscle resistance to passive movement cannot be directly associated with spasticity unless combined with neurophysiological measurement and vice versa^8^. Accordingly, ordinal measures have been proposed as valid clinical assessment tools for the evaluation of abnormal muscle resistance to passive motion, which has a partial reflexiogenic origin^12^.

On the contrary, the Myotonometer^a^ seems to discriminate between participants with and without stroke when changes in muscle compliance during muscle contraction are analysed^43,45^. This is consistent with previous research concluding the construct validity of using changes in muscle compliance as an indirect muscle strength measurement^48^. Hence, when differences in muscle compliance are evaluated in stroke survivors, smaller differences are considered to reflect a more spastic condition. The MyotonPRO^b^ scores also discriminate between stroke patients and control participants when muscle stiffness of the gastrocnemius medialis is evaluated^42^. Similarly, the MytonPRO also demonstrates to have a sufficient ability to discriminate between participants with and without paratonia^46^. Taking into account these results, the myotonometric devices seem to be a valid discriminative tool for the assessment of muscle viscoelastic property assessment in patients after stroke. However, this should be cautiously considered due to the small number of studies included. Well-known differences for validity results based on upper or lower limb muscles or type of myotonometric device were not observed. Additional research is required to establish a more solid discriminative pattern.

For reliability studies, results from meta-analysis reported excellent intra-rater reliability (ICC > 0.75) for muscle tone, elasticity and stiffness assessment in the upper limbs. Although it was not possible to pool the findings for the evaluation of muscle stiffness and elasticity of the lower limbs, a moderate to excellent intra-rater reliability was observed for all of these parameters. Similar relative reliability coefficients were obtained by Ko et al.^49^ when assessing lower limb muscle properties in spinal cord injury patients. The present findings are also consistent with those reported for non-neurological diseases^50,51^.

In general, intra-rater reliability was lower in the lower limbs than in the upper limbs. Similar results were reported for the overall intra and inter-rater reliability of the MAS^52^. It seems that the length and the weight of the lower limbs may hamper the assessment process. Two important issues need to be considered with regards to the reliability of the myotonometric devices. First, only the Myoton-3^b^ and the MyotonPRO^b^ were used for reliability analysis. Second, inter-rater reliability of myotonometry has been scarcely investigated. In stroke patients, myotonometry shows moderate to excellent ICCs for all parameters in the evaluation of the upper limbs^38^. Very similar coefficients were reported in the muscles around the shoulder after breast cancer surgery^51^. Yet, inter-rater reliability in patients with paratonia is noticeably lower, which could be explained by the inherent variability of this type of muscle tone^46^. Finally, we observed a good absolute reliability (SEM 2–15.1%), which is congruent with previous research^46,51^. In summary, it can be concluded that myotonometric devices are highly reliable for assessing muscle viscoelastic properties in stroke patients, although new research focusing on inter-rater reliability is warranted.

There is compelling evidence suggesting that the assessment of the spastic hypertonia syndrome after stroke remains a challenge. Aloraini et al.^10^ conducted a systematic review aiming to appraise the most frequent clinical measures of spasticity after stroke reported by the literature. They summarized the psychometric properties of 15 tools (instrumented and non-instrumented) and concluded a need for objective clinical tools for the evaluation of this condition. Furthermore, the authors encouraged future research to focus on investigating the psychometric properties of clinical measures of spasticity that can be easily interpreted by clinicians. For more objective tools, particularly those that quantitatively assess spasticity by recording biomechanical and electrical signals during muscle stretching, it is concluded that no method has been sufficiently assessed on all psychometric properties. Nevertheless, these tools are more advisable than the isolated use of clinical scales^47^. Thus, it seems clear that no single measure should be used alone to assess the spastic condition^16^. With this in mind, myotonometric devices could be valid to identify those mechanical muscle properties associated with the spastic hypertonia syndrome.

Assessment protocols should be conducted under different muscle conditions (e.g., relaxation, contraction and stretching at different velocities) in order to be congruent with traditional^5^ and updated definitions of spasticity^16^. During the assessment, it would be necessary to take into account important variables, such as the patient and muscle position, the number of measurement repetitions, right-left side in case of bilateral evaluation, the presence of pain and/or fear or restiveness, contextual factors, e.g., temperature, noise, and the evaluation of different muscle spots located in the muscle belly, but also in the muscle tendon. After that, the myotonometric variables should be interpreted in combination with biomechanical measures, so that a distinction between neural and non-neural spasticity components can be achieved, and with the use of function scales^12^. The collection of different data would help clinicians to identify different aspects of the spastic condition, as a multifactorial phenomenon.

This study has some limitations. First, different types of myotonometric devices with different mechanisms were analyzed. Although scores from different devices are correlated, particularly during active muscle contraction, this was only studied in young healthy males^53^. Second, spasticity-related muscle adaptations, both in the upper^54^ and lower extremities^55^, may differ during different post stroke stages, although there is no clear evidence on this issue. In our review, only one research group investigated acute stroke patients^37,38^ and a single study included subacute stroke participants^39^, which made it difficult and no relevant to perform a subgroup analysis. Overall, findings about reliability were moderate to high for upper and lower limbs in all post-stroke stages, which is important to guide proper clinical decisions.

Third, the heterogeneity of the reference standard used in the validation studies made it impossible to carry out a concurrent validity meta-analysis. Finally, we noticed that some studies referred to the same sample population^37,38,40,41^. Some repeated information from these studies has only been considered once.

## Conclusions

Myotometry seems to be a valid and reliable complementary tool when assessing muscle viscoelastic properties in stroke survivors. It is relatively easy and quick to administer, highly objective, and the devices are portable. The clinical interpretation of the different muscle parameters could help to quantify the spastic post-stroke condition.

Future research should focus on the validation of myotonometric devices using biomechanical and neurophysiological measurements as reference standards and analyse the inter-rater absolute and relative reliability for upper and lower limbs assessment. It would also be advisable to compare the psychometric properties of the different myotonometric devices between acute, subacute and chronic stroke patients. Finally, these studies need to provide reports on clear and reproducible protocols.

## Supplementary Information

Supplementary Information
