# Supplementary material for: Validity and reliability of myotonometry for assessing muscle viscoelastic properties in patients with stroke: a systematic review and meta-analysis
Source: Sci Rep. 2021 Mar 3;11:5062. doi: 10.1038/s41598-021-84656-1 (PMC7930253; doi:10.1038/s41598-021-84656-1)
Supplement: Supplementary file 1 — Supplementary Information [file 41598_2021_84656_MOESM1_ESM.docx]

Validity and reliability of myotonometry for assessing muscle viscoelastic properties in patients with stroke: A systematic review and meta-analysis

Maria-Isabel Garcia-Bernal, PT ^a^; Alberto Marcos Heredia-Rizo, PT, PhD ^a^; Paula Gonzalez-Garcia, PT, PhD ^a^*, María Dolores Cortés-Vega PT, PhD ^a^, María Jesús Casuso-Holgado, PT, PhD ^a^

Supplementary table S1. Detailed search strategy.

| **Nº term used** |
| --- |
| **#1** “Musc* tone”  **#2** Spastic*  **#3** “Musc* stiffness”  **#4** “Musc* Propert*”  **#5** Valid*  **#6** Reliab*  **#7** Reproducib*  **#8** Accurary  **#9** Psychometric  **#10** Stroke  **#11** “Brain Injury”  **#12** “Cerebrovascular accident”  **#13** Hemipleg*  **#14** Hemipares*  **#15** CVA  **#16** Apoplex* |

**PUBMED (216 potential articles):**

((MUSC* TONE) OR SPATIC* OR (MUSC* STIFFNESS) OR (MUSC* PROPERT*)) AND (VALID* OR RELIAB* OR REPRODUCIB* OR ACCURA* OR PSYCHOMETR*) AND (STROKE OR (BRAIN INJURY) OR (CEREBROVASCULAR ACCIDENT*) OR HEMIPLEG* OR HEMIPARES* OR CVA OR APOPLEXY

**SCOPUS (465 potential articles):**

( TITLE-ABS-KEY ( ( MUSC* AND TONE ) OR SPATIC* OR ( MUSC* AND STIFFNESS ) OR ( MUSC* AND PROPERT* ) ) AND TITLE-ABS-KEY ( VALID* OR RELIAB* OR REPRODUCIB* OR ACCURA* OR PSYCHOMETR* ) AND TITLE-ABS-KEY ( STROKE OR ( BRAIN AND INJURY ) OR ( CEREBROVASCULAR AND ACCIDENT* ) OR HEMIPLEG* OR HEMIPARES* OR CVA OR APOPLEXY ) )

**CINAHL (126 potential articles):**

#1 OR #2 OR #3 OR #4 AND #5 OR #6 OR #7 OR #8 OR #9 AND #10 OR #11 OR #12 OR #13 OR #14 OR #15 OR #16

**PEDRO (4 potential articles)**

#1 AND #10 (3 potential articles)

#2 and #10 (1 potential articles)

**Note: only strategies with results are showed.**

**Supplementary Table 2.** QUADAS-2 data extraction score.

| **Studies** | **Domain 1: patient selection**  **Bias/applicability** | **Domain 2: index test**  **Bias/applicability** | **Domain 3:reference standard**  **Bias/applicability** | **Domain 4: flow and timing**  **Bias** |
| --- | --- | --- | --- | --- |
| Lo et al. ^(37)^ | Low/Low | N/A | N/A | N/A |
| Lo et al. ^(38)^ | Low/Low | N/A | N/A | N/A |
| Chuang et al. ^(39)^ | High/Unclear | N/A | N/A | N/A |
| Chuang et al. ^(40)^ | High/Unclear | Low/Low | High/High | Low |
| Chuang et al. ^(41)^ | High/Unclear | N/A | N/A | N/A |
| Fröhlich-Zwahlen et al. ^(42)^ | Low/Low | Low/Low | High/Unclear | Unclear |
| Leonard et al. ^(43)^ | Unclear/Unclear | Low/Low | Unclear/Low | Low |
| Li et al. ^(44)^ | Low/Low | Low/Low | Unclear/Low | Low |
| Rhydal & Brouwer^(45)^ | Low/Low | Unclear/Low | Low/Low | Low |

N/A: not applicable for non-criterion validity studies.

**Supplementary Table 3**. COSMIN critical appraisal tool data extraction score.

| **Study** | **Reliability**  **(Box 6)** | **Measurement error**  **(Box 7)** | **Criterion validity**  **(Box 8)** | **Construct validity**  **(Box9)** | **Responsiveness**  **(Box 10)** |
| --- | --- | --- | --- | --- | --- |
| Lo et al. ^(37)^ | Doubtful | Doubtful | N/A | N/A | N/A |
| Lo et al. ^(38)^ | Doubtful | Doubtful | N/A | N/A | N/A |
| Chuang et al.^(39)^ | Doubtful | Doubtful | N/A | N/A | N/A |
| Chuang et al. ^(40)^ | Doubtful | Inadequate | Doubtful | N/A | Inadequate |
| Chuang et al.^(41)^ | Doubtful | Doubtful | N/A | N/A | N/A |
| Fröhlich-Zwahlen et al.^(42)^ | Inadequate | Inadequate | Very good | Doubtful | N/A |
| Leonard et al.^(43)^ | N/A | N/A | Inadequate | Inadequate | N/A |
| Li et al.^(44)^ | N/A | N/A | Very good | N/A | N/A |
| Rhydal & Brouwer^(45)^ | N/A | N/A | Doubtful | Adequate | N/A |

Abbreviations: N/A: not applicable

**Table S4.** Methodological quality by COSMIN checklist of the studies analyzing reliability.

| Questions | Studies | Lo et al. ^(37)^ | Lo et al. ^(38)^ | Chuang et al.^(39)^ | Chuang et al. ^(40)^ | Chuang et al.^(41)^ | Fröhlich-Zwahlen et al.^(42)^ | Leonard et al.^(43)^ | Li et al.^(44)^ | Rhydal & Brouwer^(45)^ |
| --- | --- | --- | --- | --- | --- | --- | --- | --- | --- | --- |
| Were patients stable in the interim period on the construct to be measured? | | D | A | A | A | A | D | N/A | N/A | N/A |
| Was the time interval appropriate? | | D | VG | VG | VG | VG | I | N/A | N/A | N/A |
| Were the test conditions similar for the measurements? e.g. type of administration, environment, instructions | | VG | VG | VG | VG | VG | A | N/A | N/A | N/A |
| For continuous scores: Was an intraclass correlation coefficient (ICC) calculated? | | VG | VG | VG | VG | VG | VG | N/A | N/A | N/A |
| For dichotomous/nominal/ordinal scores: Was kappa calculated? | | N/A | N/A | N/A | N/A | N/A | N/A | N/A | N/A | N/A |
| For ordinal scores: Was a weighted kappa calculated? | | N/A | N/A | N/A | N/A | N/A | N/A | N/A | N/A | N/A |
| For ordinal scores: Was the weighting scheme described? e.g. linear, quadratic | | N/A | N/A | N/A | N/A | N/A | N/A | N/A | N/A | N/A |
| Were there any other important flaws in the design or statistical methods of the study? | | D | D | D | D | D | D | N/A | N/A | N/A |
| Rating | | D | D | D | D | D | I | N/A | N/A | N/A |

**AD**: adequate, **D**: doutbtfoul, **I**: inadequate, **N/A**: not applicable, **VG**: very good.

**Table S5.** Methodological quality by COSMIN checklist of the studies analyzing reliability measurement error.

| Questions | Studies | Lo et al. ^(37)^ | Lo et al. ^(38)^ | Chuang et al.^(39)^ | Chuang et al. ^(40)^ | Chuang et al.^(41)^ | Fröhlich-Zwahlen et al.^(42)^ | Leonard et al.^(43)^ | Li et al.^(44)^ | Rhydal & Brouwer^(45)^ |
| --- | --- | --- | --- | --- | --- | --- | --- | --- | --- | --- |
| Were patients stable in the interim period on the construct to be measured? | | D | A | A | A | A | D | N/A | N/A | N/A |
| Was the time interval appropriate? | | D | VG | VG | VG | VG | I | N/A | N/A | N/A |
| Were the test conditions similar for the measurements? (e.g. type of administration, environment, instructions | | VG | VG | VG | VG | VG | A | N/A | N/A | N/A |
| For continuous scores: Was the Standard Error of Measurement (SEM), Smallest Detectable Change (SDC) or Limits of Agreement (LoA) calculated? | | VG | VG | VG | I | VG | VG | N/A | N/A | N/A |
| For dichotomous/nominal/ordinal scores: Was the percentage (positive and negative) agreement calculated? | | N/A | N/A | N/A | N/A | N/A | N/A | N/A | N/A | N/A |
| Were there any other important flaws in the design or statistical methods of the study? | | D | D | D | D | D | D | N/A | N/A | N/A |
| Rating | | D | D | D | I | D | I | N/A | N/A | N/A |

**AD**: adequate, **D**: doutbtfoul, **I**: inadequate, **N/A**: not applicable, **VG**: very good.

**Table S6.** Methodological quality by COSMIN checklist of the studies analyzing criterion validity.

| Questions | Studies | Lo et al. ^(37)^ | Lo et al. ^(38)^ | Chuang et al.^(39)^ | Chuang et al. ^(40)^ | Chuang et al.^(41)^ | Fröhlich-Zwahlen et al.^(42)^ | Leonard et al.^(43)^ | Li et al.^(44)^ | Rhydal & Brouwer^(45)^ |
| --- | --- | --- | --- | --- | --- | --- | --- | --- | --- | --- |
| For continuous scores: Were correlations, or the area under the receiver operating curve calculated? | | N/A | N/A | N/A | VG | N/A | VG | VG | VG | VG |
| For dichotomous scores: Were sensitivity and specificity determined? | | N/A | N/A | N/A | N/A | N/A | N/A | N/A | N/A | N/A |
| Were there any other important flaws in the design or statistical methods of the study? | | N/A | N/A | N/A | D | N/A | VG | I | VG | D |
| Rating | | N/A | N/A | N/A | D | N/A | VG | I | VG | D |

**AD**: adequate, **D**: doubtful, **I**: inadequate, **N/A**: not applicable, **VG**: very good.

**Table S7.** Methodological quality by COSMIN checklist of the studies analyzing construct validity.

| Questions | Studies | Lo et al. ^(37)^ | Lo et al. ^(38)^ | Chuang et al.^(39)^ | Chuang et al. ^(40)^ | Chuang et al.^(41)^ | Fröhlich-Zwahlen et al.^(42)^ | Leonard et al.^(43)^ | Li et al.^(44)^ | Rhydal & Brouwer^(45)^ |
| --- | --- | --- | --- | --- | --- | --- | --- | --- | --- | --- |
| Is it clear what the comparator instrument(s) measure(s)? | | N/A | N/A | N/A | N/A | N/A | VG | VG | N/A | VG |
| Were the measurement properties of the comparator instrument(s) sufficient? | | N/A | N/A | N/A | N/A | N/A | D | VG | N/A | VG |
| Was the statistical method appropriate for the hypotheses to be tested? | | N/A | N/A | N/A | N/A | N/A | VG | D | N/A | VG |
| Were there any other important flaws in the design or statistical methods of the study? | | N/A | N/A | N/A | N/A | N/A | D | I | N/A | VG |
| Was an adequate description provided of important characteristics of the subgroups? | | N/A | N/A | N/A | N/A | N/A | VG | D | N/A | A |
| Was the statistical method appropriate for the hypotheses to be tested? | | N/A | N/A | N/A | N/A | N/A | VG | D | N/A | VG |
| Were there any other important flaws in the design or statistical methods of the study? | | N/A | N/A | N/A | N/A | N/A | VG | I | N/A | VG |
| Rating | | N/A | N/A | N/A | N/A | N/A | D | I | N/A | A |

**AD**: adequate, **D**: doutbtfoul, **I**: inadequate, **N/A**: not applicable, **VG**: very good.

**Table S8.** Methodological quality by COSMIN checklist of the studies analyzing responsiveness**.**

| Questions | Studies | Lo et al. ^(37)^ | Lo et al. ^(38)^ | Chuang et al.^(39)^ | Chuang et al. ^(40)^ | Chuang et al.^(41)^ | Fröhlich-Zwahlen et al.^(42)^ | Leonard et al.^(43)^ | Li et al.^(44)^ | Rhydal & Brouwer^(45)^ |
| --- | --- | --- | --- | --- | --- | --- | --- | --- | --- | --- |
| For continuous scores: Were correlations between change scores, or the area under the Receiver Operator Curve (ROC) curve calculated? | | N/A | N/A | N/A | I | N/A | N/A | N/A | N/A | N/A |
| For dichotomous scales: Were sensitivity and specificity (changed versus not changed) determined? | | N/A | N/A | N/A | N/A | N/A | N/A | N/A | N/A | N/A |
| Were there any other important flaws in the design or statistical methods of the study? | | N/A | N/A | N/A | I | N/A | N/A | N/A | N/A | N/A |
| Is it clear what the comparator instrument(s) measure(s)? | | N/A | N/A | N/A | VG | N/A | N/A | N/A | N/A | N/A |
| Were the measurement properties of the comparator instrument(s) sufficient? | | N/A | N/A | N/A | A | N/A | N/A | N/A | N/A | N/A |
| Was the statistical method appropriate for the hypotheses to be tested? | | N/A | N/A | N/A | A | N/A | N/A | N/A | N/A | N/A |
| Were there any other important flaws in the design or statistical methods of the study? | | N/A | N/A | N/A | I | N/A | N/A | N/A | N/A | N/A |
| Was an adequate description provided of important characteristics of the subgroups? | | N/A | N/A | N/A | N/A | N/A | N/A | N/A | N/A | N/A |
| Was the statistical method appropriate for the hypotheses to be tested? | | N/A | N/A | N/A | N/A | N/A | N/A | N/A | N/A | N/A |
| Were there any other important flaws in the design or statistical methods of the study? | | N/A | N/A | N/A | N/A | N/A | N/A | N/A | N/A | N/A |
| Was an adequate description provided of the intervention given? | | N/A | N/A | N/A | I | N/A | N/A | N/A | N/A | N/A |
| Was the statistical method appropriate for the hypotheses to be tested? | | N/A | N/A | N/A | D | N/A | N/A | N/A | N/A | N/A |
| Were there any other important flaws in the design or statistical methods of the study? | | N/A | N/A | N/A | I | N/A | N/A | N/A | N/A | N/A |
| Rating | | N/A | N/A | N/A | I | N/A | N/A | N/A | N/A | N/A |

**AD**: adequate, **D**: doutbtfoul, **I**: inadequate, **N/A**: not applicable, **VG**: very good.
